# Supplementary material for: Genotypic Difference in the Responses to Nitrogen Fertilizer Form in Tibetan Wild and Cultivated Barley
Source: Plants (Basel). 2021 Mar 22;10(3):595. doi: 10.3390/plants10030595 (PMC8004229; doi:10.3390/plants10030595)
Supplement: Supplementary file 1 [file plants-10-00595-s001.pdf]

Table S1. Modified barley basal nutrient solution composition

Stock	Treat-1 (NO_3^-)	Treat-2 (NH_4^+)	Treat-3 (Urea)	Treat-4 (Glycine)	(g/L)	Stock Conc.	Final Conc.	10 L	30L
H1*	KNO_3 (101.1 g/mol)				101.1 g/L	1M	2 mM	20ml	60ml
		$(NH_4)_2SO_4$ (MW 132.14 g/mol)			132.14g/L	1M	1mM	10ml	30ml
			CH_4N_2O MW (60.1 g/mol)		60.1 g/L	1M	1 mM	10ml	30ml
				$C_2H_5NO_2$ MW (75.07 g/mol)	75.07 g/L	1M	2 mM	20ml	60ml
		KCl MW (74.55 g/mol)	KCl MW (74.55 g/mol)	KCl MW (74.55 g/mol)	74.55 g/L	1M	2 mM	20ml	60ml
	$CaCl_2 \cdot 2H_2O$ MW (147.0 g/mol)				147 g/L	1M	0.9 mM	9 ml	27ml
H2	$Ca(H_2PO_4)_2$ MW (234.05 g/mol)				234.05 g/L	1M	0.1 mM	1 ml	3ml
		$Ca(H_2PO_4)_2$ MW (234.05 g/mol)	$Ca(H_2PO_4)_2$ MW (234.05 g/mol)	$Ca(H_2PO_4)_2$ MW (234.05 g/mol)	234.05 g/L	1M	1 mM	10ml	30ml
H3	$MgSO_4.7H_2O$ MW (246.46)	$MgSO_4.7H_2O$ MW (246.46)	$MgSO_4.7H_2O$ MW (246.46)	$MgSO_4.7H_2O$ MW (246.46)	246.46 g/L	1M	0.4 mM	4 ml	12ml
H4	H_3BO_3 MW (61.83 g/mol)	H_3BO_3 MW (61.83 g/mol)	H_3BO_3 MW (61.83 g/mol)	H_3BO_3 MW (61.83 g/mol)	0.185 g/L	0.003 M	3 uM	10ml	30ml
	$MnCl_2 \cdot 4H_2O$ MW (197.91 g/mol)	$MnCl_2 \cdot 4H_2O$ MW (197.91 g/mol)	$MnCl_2 \cdot 4H_2O$ MW (197.91 g/mol)	$MnCl_2 \cdot 4H_2O$ MW (197.91 g/mol)	0.99 g/L	0.005 M	0.5 uM		
	$Na_2MoO_4 \cdot 2H_2O$ MW (241.95 g/mol)	$Na_2MoO_4 \cdot 2H_2O$ MW (241.95 g/mol)	$Na_2MoO_4 \cdot 2H_2O$ MW (241.95 g/mol)	$Na_2MoO_4 \cdot 2H_2O$ MW (241.95 g/mol)	0.242 g/L	0.001 M	1 uM		
	$ZnSO_4 \cdot 7H_2O$ MW (287.56 g/mol)	$ZnSO_4 \cdot 7H_2O$ MW (287.56 g/mol)	$ZnSO_4 \cdot 7H_2O$ MW (287.56 g/mol)	$ZnSO_4 \cdot 7H_2O$ MW (287.56 g/mol)	1.15 g/L	0.004 M	0.4 uM		
H5	$CuSO_4 \cdot 5H_2O$ MW (249.68 g/mol)	$CuSO_4 \cdot 5H_2O$ MW (249.68 g/mol)	$CuSO_4 \cdot 5H_2O$ MW (249.68g/mol)	$CuSO_4 \cdot 5H_2O$ MW (249.68 g/mol)	0.25 g/500 ml	0.002 M	0.2 uM	10ml	30ml
H6	Fe(III)Na-EDTA MW (367.15g/mol))	Fe(III)Na-EDTA MW (367.15g/mol)	Fe(III)NaEDTA MW (367.15g/mol)	Fe(III)Na-EDTA MW (367.15g/mol)	7.343 g/L	0.02 M	20 uM	10ml	30ml

*H1 have 4 forms of nitrate, H2 have different conc., H3,H4,H5,H6 are the common stock, MW= Molecular Weight
